# Outcome Comparison of High-Risk Native American Patients Who Did or Did Not Receive Monoclonal Antibody Treatment for COVID-19

**DOI:** 10.1001/jamanetworkopen.2021.25866

**Published:** 2021-09-21

**Authors:** Ryan M. Close, T. Shaifer Jones, Christopher Jentoft, James B. McAuley

**Affiliations:** 1 Whiteriver Indian Hospital, Indian Health Service, Whiteriver, Arizona

## Abstract

This quality improvement study compares outcomes among high-risk Native American patients who did or did not receive monoclonal antibody treatment for COVID-19.

## Introduction

Treatments for COVID-19 remain urgent and necessary despite increasing vaccine distribution. Studies suggest that monoclonal antibody (mAb) therapies prevent progression in early disease.^1,2^ In late 2020, the US Food and Drug Administration (FDA) issued an emergency use authorization (EUA) for 2 mAb therapies, bamlanivimab and a combination of casirivimab and imdevimab, to treat COVID-19.^3,4^ However, previous mAb studies^1,2^ with a total of 852 participants (577 in one study^1^ and 275 in the other^2^) did not report a reduction in patient mortality, and only 5 participants across both trials (0.6%) were Native American. We present a retrospective quality improvement study on an early mAb treatment program for high-risk Native American patients at the Whiteriver Service Unit (WRSU), a rural acute care facility that serves as the primary hospital and public health department on the Fort Apache Indian Reservation in eastern Arizona.

## Methods

For this quality improvement study, all WRSU patients who had a positive COVID-19 test result during the observation period (between December 1, 2020, and February 3, 2021) were screened for mAb treatment eligibility per the EUA. All eligible patients provided oral informed consent. Patients were treated with bamlanivimab or a combination of casirivimab and imdevimab according to manufacturer and FDA guidelines^3,4^ and monitored for 30 days. Post hoc exploratory analyses compared mAb-treated patients with patients with COVID-19 who met the EUA high-risk criteria but were not treated for various reasons. See the eMethods in the Supplement for additional details. The Tribal Health Board and White Mountain Apache Tribal Council approved the study procedures and their publication. The study followed the Standards for Quality Improvement Reporting Excellence (SQUIRE) reporting guideline.

## Results

During the observation period, 983 WRSU patients received a positive COVID-19 test result. The median patient age was 32 years (interquartile range [IQR], 17-51 years) and 534 patients (54.3%) were female. Of the 983 patients, 481 (48.9%) met EUA high-risk criteria for treatment and 201 high-risk patients (41.8%) received mAb treatment. The median time from COVID-19 test collection to mAb treatment was 23 hours (IQR, 3-45 hours), and 182 of 201 patients (90.5%) received treatment within 72 hours. The median time from symptom onset to treatment was 2 days (IQR, 1-3 days), and 113 of 149 symptomatic patients (75.8%) were treated within 3 days (Table 1). The mAb-treated patients had a median body mass index (calculated as weight in kilograms divided by height in meters squared) of 35.8 (IQR, 30-40) and a mean (SD) age of 50 (19) years, and 114 (56.7%) met 2 or more high-risk criteria. The mAb-treated patients were older and had more risk factors for severe disease than nonrecipients (Table 1). The 280 high-risk nonrecipients had a mean (SD) age of 43 (19) years, and 125 (44.6%) met 2 or more high-risk criteria. Compared with nonrecipients, the mAb-treated patients had a lower proportion of acute medical visits (59 [29.4%] vs 136 [48.6%]), hospitalizations (35 [17.4%] vs 120 [42.9%]), transfers to outside facilities (4 [2%] vs 26 [9.3%]), intensive care unit admissions (0 vs 12 [4.3%]), and deaths (0 vs 8 [2.9%]) (Table 2). Of the 8 deaths during the observation period, these patients all met the EUA high-risk criteria but did not receive mAb treatment.

**Table 1.** Demographic Comparison of Patients Who Did or Did Not Receive Monoclonal Antibody Treatment for COVID-19

Characteristic	No. (%)		*P* value
	mAb recipients (n = 201)	Nonrecipients (n = 280)	
Native American ethnicity	201 (100)	280 (100)	NA
Sex			
Female	123 (61.2)	151 (53.9)	.11
Male	78 (38.8)	129 (46.1)	
Age			
Mean (SD), y	50 (19)	43 (19)	<.001
≥65 y	53 (26.4)	45 (16.1)	.006
BMI			
No. of patients	195	274	
Median (IQR)	35.8 (30.0-40.0)	35.0 (29.2-38.4)	.15
≥35^a^	118 (60.5)	163 (59.5)	.82
Comorbidities^b^			
Chronic kidney disease	14 (7.0)	14 (5.0)	.36
Diabetes	85 (42.3)	112 (43.57)	.78
Immunosuppressive disease	2 (1.0)	1 (0.4)	.38
Immunosuppressive treatment	6 (3.0)	5 (1.8)	.39
Chronic respiratory disease	39 (19.4)	42 (16.8)	.46
Aged ≥55 y with the following:			
Hypertension	62 (30.9)	62 (22.1)	.03
Cardiovascular disease	18 (9.0)	18 (6.4)	.30
Other^c^	1 (0.5)	9 (3.2)	.05
Risk factors for severe COVID-19, No.^b^			
Median (IQR)	2 (1-3)	1 (1-2)	.007
≥1	200 (99.5)	280 (100)	.24
≥2	114 (56.7)	125 (44.6)	.009
Treatment			
Bamlanivimab	180 (89.6)	NA	NA
Combination of casirivimab and imdevimab	21 (10.4)	NA	NA
Test collection to treatment, median (IQR), h	23 (3-45)	NA	NA
Symptom onset to treatment, median (IQR), d^d^	2 (1-3)	NA	NA

Abbreviations: BMI, body mass index (calculated as weight in kilograms divided by height in meters squared); IQR, interquartile range; mAb, monoclonal antibody; NA, not applicable.

^a^ For patients aged 12 to 17 years, BMI in the 85th percentile or higher.

^b^ Risk factors based on emergency use authorization criteria were as follows: age 65 years or older, BMI (for patients aged 12-17 years, BMI in the 35th percentile or higher or in the 85th percentile or higher), chronic kidney disease, diabetes, immunosuppressive disease, immunosuppressive treatment, chronic lung disease, and age 55 years or older with hypertension or cardiovascular disease.

^c^ Other conditions included end-stage liver disease, congestive heart failure (age younger than 55 years), spina bifida, Down syndrome, or malignant neoplasm.

^d^ Among 149 patients with a known symptom onset date; 45 patients (22.4%) were asymptomatic at the time of treatment.

**Table 2.** Comparison of 30-Day Outcomes Among Patients Who Did or Did Not Receive Monoclonal Antibody Treatment for COVID-19

Outcome	mAb recipients, No. (%)	Nonrecipients, No. (%)	Point estimate, OR (95% CI)	*P* value	NNT^a^
**Among all patients**					
No. of patients	201	280			
Acute medical visit^b^	59 (29.4)	136 (48.6)	0.44 (0.29 to 0.66)	<.001	6
Emergency department visit only	24 (11.9)	16 (5.7)	NA	NA	NA
Hospitalization^c^	35 (17.4)	120 (42.9)	0.28 (0.18 to 0.44)	<.001	4
Transfer to outside facility for higher-level care	4 (2.0)	26 (9.3)	0.20 (0.05 to 0.59)	.001	14
Intensive care unit admission	0	12 (4.3)	−4.3 (−6.7 to −1.9)^d^	.003	24
Death	0	8 (2.9)^c^	−2.9 (−4.8 to −0.9)^d^	.008	35
Adverse reaction	1 (0.5)	NA	NA	NA	NA
**Among hospitalized patients**					
No. of patients	35	120			
Symptom duration at admission, No./total No. (%)					
Asymptomatic	2/35 (6)	5/120 (4)	1.4 (0.13 to 9)	.66	NA
Days, median (IQR)^e^	6 (3-9)	5 (3-8)		.66	NA
Admission in ≤3 d^e^	10/32 (31)	35/108 (32)	0.95 (0.36 to 2.4)	.90	NA
Days in hospital, median (IQR)	4 (3-5)	4 (4-5)		.48	NA
Oxygen requirement, No./total No. (%)	26/35 (74)	91 (76)	0.92 (0.36 to 2.5)	.53	NA
None	9/35 (26)	29/120 (24)	NA	.54	NA
Low flow	25/35 (71)	81/120 (68)			
NRB mask, high flow, or BiPAP	1/35 (3)	4/120 (3)			
Intubation	0/35	6/120 (5)			

Abbreviations: BiPAP, bilevel positive airway pressure; IQR, interquartile range; mAb, monoclonal antibody; NA, not applicable; NNT, number needed to treat; NRB, nonrebreather; OR, odds ratio.

^a^ Number needed to treat to prevent the given medical outcome. Only given if *P* < .05.

^b^ COVID-19–related emergency department visit or hospitalization.

^c^ COVID-19–related hospitalization, including local hospitalizations and transfers.

^d^ Absolute risk reductions are given as percentages when ORs were not possible.

^e^ Among patients with known symptom onset dates (32 mAb-treated patients and 108 nontreated patients).

## Discussion

The lower proportion of hospitalizations among mAb-treated patients observed at WRSU is consistent with previous mAb studies,^1,2^ albeit to a greater degree. To our knowledge, this study is the first to report mAb use for Native American individuals and is among the few studies to report a significantly lower proportion of deaths among patients receiving mAbs. The magnitude of our findings is attributable in part to a higher baseline hospitalization rate. Abiding by EUA criteria meant that eligible WRSU patients, treated and untreated, were at higher risk for severe COVID-19 (eg, higher BMI and more risk factors) compared with prior studies.^1,2^ Furthermore, Native American individuals experience greater infectious disease mortality, including from COVID-19, which likely increased WRSU hospitalization rates.^5,6^ The success of the WRSU mAb program is also likely attributable to rapid treatment. The WRSU decreased the time to treatment by integrating contact tracing, clinical outreach, in-house molecular testing, and a unified public health and hospital system that streamlined information exchange. This approach may not be generalizable but is a model for other centralized health systems.

We recognize several limitations of this study. Retrospective comparisons can result in some misclassification. In particular, some patients in this study were excluded from mAb treatment solely based on symptom severity, per the EUA.^3,4^ These patients may have progressed to hospitalization even with mAb treatment; however, it is also possible that earlier mAb intervention may have prevented this progression. Given the magnitude of our findings, we believe they are unlikely to be entirely the result of misclassification. Therefore, we assert that the success of the WRSU mAb program suggests that these treatments can be used to great effect in rural, relatively resource-limited settings.

## Notes

Supplement. **eMethods.** Supplemental Methods
