# Supplementary material for: Outcome Comparison of High-Risk Native American Patients Who Did or Did Not Receive Monoclonal Antibody Treatment for COVID-19
Source: JAMA Netw Open. 2021 Sep 21;4(9):e2125866. doi: 10.1001/jamanetworkopen.2021.25866 (PMC8456379; doi:10.1001/jamanetworkopen.2021.25866)
Supplement: Supplement. — eMethods. Supplemental Methods [file jamanetwopen-e2125866-s001.pdf]

# Supplementary Online Content

Close RM, Jones TS, Jentoft C, McAuley JB. Outcome comparison of high-risk Native American patients who did or did not receive monoclonal antibody treatment for COVID-19. *JAMA Netw Open*. 2021;4(9):e2125866. doi:10.1001/jamanetworkopen.2021.25866

**eMethods.** Supplemental Methods

This supplementary material has been provided by the authors to give readers additional information about their work.

© 2021 Close RM et al. *JAMA Network Open*.

## eMethods. Supplemental methods

Beginning on December 1st, all patients testing positive for SARS-CoV-2 via molecular testing at WRSU were screened for MAb treatment eligibility according to FDA EUA criteria. This study is limited to patients who tested positive between December 1, 2020 and February 3, 2021. Eligible patients were required to have at least one risk factor for severe COVID-19 per EUA (age ≥ 65, BMI ≥ 35 or ≥ 85th percentile for patients 12-17 years old, chronic kidney disease, diabetes mellitus, immunosuppressive disease, immunosuppressive treatment, chronic lung disease, age ≥ 55 with hypertension or cardiovascular disease, or other significant comorbidity). Eligible patients were further evaluated by clinicians to determine symptom severity and time course of illness, and patients requiring hospitalization were excluded (per EUA). All eligible patients were informed of EUA and consented prior to infusion and were treated with either bamlanivimab or casirivimab/imdevimab, the only approved MAb therapies at the time of the study, according to manufacturer and FDA guidelines. Background data on eligible patients was collected through a chart review, including age, gender, interval from symptom onset to infusion, interval from test collection to infusion, oxygen saturation and symptoms at time of infusion. Risk factors for severe COVID-19 were enumerated based on EUA and other established variables, as listed above. Patients were followed for 30 days after infusion (treated) or test result (untreated), and data was collected on medical outcomes including emergency room visits, hospitalizations, transfers to higher levels of care, ICU admissions, lengths of hospital stays, peak inpatient oxygen requirements, and deaths.

Since the primary objective was to evaluate and improve our process for administering MAb therapies and there was no control group, there were no sample size or power calculations. Post-hoc exploratory analyses compared MAb-treated patients with patients from the same community who tested positive for COVID-19 at WRSU during the same period and met high-risk EUA criteria but did not receive treatment (refused, medication unavailable, missed opportunity, severity of illness at diagnosis, etc.). Due to incomplete data, totals and proportions are not available for each of the reasons for non-treatment, but supply was limited to < 20 doses of MAb treatments per week at the beginning of the program via the National Supply Service Center. Descriptive and comparative statistics were computed using parametric and non-parametric methods where appropriate and all analyses were performed using STATA/IC, version 16.1 (StataCorp). We adhered to SQUIRE 2.0 guidelines for quality improvement studies.

© 2021 Close RM et al. *JAMA Network Open*.
